# Risk factors of unintentional piecemeal resection in endoscopic mucosal resection for colorectal polyps ≥ 10 mm

**DOI:** 10.1038/s41598-023-50815-9

**Published:** 2024-01-04

**Authors:** Tsubasa Ishikawa, Kenichiro Okimoto, Tomoaki Matsumura, Sadahisa Ogasawara, Yoshihiro Fukuda, Yoshio Kitsukawa, Yuya Yokoyama, Kengo Kanayama, Naoki Akizue, Yotaro Iino, Yuki Ohta, Hideaki Ishigami, Takashi Taida, Shin Tsuchiya, Keiko Saito, Hidehiro Kamezaki, Akitoshi Kobayashi, Yasuharu Kikuchi, Minoru Tada, Yuki Shiko, Yoshihito Ozawa, Jun Kato, Taketo Yamaguchi, Naoya Kato

**Affiliations:** 1 Department of Gastroenterology, Graduate School of Medicine, Chiba University, Inohana 1-8-1, Chuo-ku, Chiba-City, 260-8670 Japan; 2 Translational Research and Development Center, Chiba University Hospital, Chiba, Japan; 3 Department of Gastroenterology, Seikei-kai Chiba Medical Center, Chiba, Japan; 4 Department of Gastroenterology, Chiba Municipal Aoba Hospital, Chiba, Japan; 5 Department of Gastroenterology, Chibaken Saiseikai Narashino Hospital, Chiba, Japan; 6 Department of Gastroenterology, Kimitsu Chuo Hospital, Chiba, Japan; 7 Department of Gastroenterology, Chiba Rosai Hospital, Chiba, Japan; 8 Department of Gastroenterology, Funabashi Central Hospital, Chiba, Japan; 9 Department of Gastroenterology, Eastern Chiba Medical Center, Chiba, Japan; 10 Department of Gastroenterology, Funabashi Municipal Medical Center, Chiba, Japan; 11 Department of Gastroenterology, Numazu City Hospital, Shizuoka, Japan; 12 Department of Gastroenterology, National Hospital Organization Chiba Medical Center, Chiba, Japan; 13 Biostatistics Section, Clinical Research Center, Chiba University Hospital, Chiba, Japan

**Keywords:** Colonoscopy, Gastrointestinal diseases

## Abstract

This study aimed to investigate the lesion and endoscopist factors associated with unintentional endoscopic piecemeal mucosal resection (uniEPMR) of colorectal lesions ≥ 10 mm. uniEPMR was defined from the medical record as anything other than a preoperatively planned EPMR. Factors leading to uniEPMR were identified by retrospective univariate and multivariate analyses of lesions ≥ 10 mm (adenoma including sessile serrated lesion and carcinoma) that were treated with endoscopic mucosal resection (EMR) at three hospitals. Additionally, a questionnaire survey was conducted to determine the number of cases treated by each endoscopist. A learning curve (LC) was created for each lesion size based on the number of experienced cases and the percentage of uniEPMR. Of 2557 lesions, 327 lesions underwent uniEPMR. The recurrence rate of uniEPMR was 2.8%. Multivariate analysis showed that lesion diameter ≥ 30 mm (odds ratio 11.83, 95% confidence interval 6.80–20.60, p < 0.0001) was the most associated risk factor leading to uniEPMR. In the LC analysis, the proportion of uniEPMR decreased for lesion sizes of 10–19 mm until 160 cases. The proportion of uniEPMR decreased with the number of experienced cases in the 20–29 mm range, while there was no correlation between the number of experienced cases and the proportion of uniEPMR ≥ 30 mm. These results suggest that 160 cases seem to be the minimum number of cases needed to be proficient in en bloc EMR. Additionally, while lesion sizes of 10–29 mm are considered suitable for EMR, lesion sizes ≥ 30 mm are not applicable for en bloc EMR from the perspective of both lesion and endoscopist factors.

## Introduction

According to 2018 data, there were 1.8 million new cases of colorectal cancer (CRC) worldwide, making it the third most common cancer among men and the second most common cancer among women globally^1^. The detection and removal of adenomatous lesions in the colon were reported to reduce the incidence and mortality of CRC by 53%^2,3^. Therefore, endoscopic treatment for colorectal lesions is now considered an important method to prevent CRC-related death.

Currently, most adenomatous colorectal lesions ≤ 9 mm are usually resected with cold polypectomy (CP)^4^. For colorectal lesions ≥ 10 mm, endoscopic mucosal resection (EMR) is the most common procedure^5,6^. In some cases, endoscopic piecemeal resection (EPMR) is performed intentionally (iEPMR), but previous studies have reported a higher local recurrence rate with EPMR than with en bloc resection^7,8^.

Although there are several reports investigating the factors of piecemeal resection, only a few reports have assessed the risk factors of unintentional EPMR (uniEPMR) in relation to the technical skills of endoscopists and features of a lesion.

Thus, this study aimed to investigate the risk factors for uniEPMR for colorectal lesions ≥ 10 mm from the viewpoint of resected lesions and endoscopist factors.

## Methods

### Study design and patients

From January 2007 to May 2018, data on EMR and hot snare polypectomy (HSP) at Chiba University Hospital, Seikei-kai Chiba Medical Center, and Chiba Municipal Aoba Hospital were collected retrospectively. At these three institutions, details of resected lesions and patient information were compiled. The inclusion criteria were lesions histopathologically diagnosed as adenoma including sessile serrated lesion or carcinoma, lesion size ≥ 10 mm, and EMR. The exclusion criteria were lesions < 10 mm, HSP or iEPMR, non-neoplastic lesions, and cases for which it could not be determined whether or not a piecemeal resection was performed. uniEPMR was defined from the medical record as anything other than a preoperatively planned EPMR. Lesion size was based on endoscopic observation. iEPMR was defined from the medical record as a preoperatively planned EPMR. Endoscopist factors, such as the number of the treatment experience for EMR and HSP for colorectal lesions, and the data for the Japan Gastroenterological Endoscopy Society (JGES) specialty were also obtained.

This study was approved by the Medical Ethics Board of Chiba University Graduate School of Medicine (protocol no.: 3593) and was conducted in accordance with the Declaration of Helsinki. The medical ethics boards of the other collaborating institutions at their respective facilities also approved this study. This was a retrospective cohort study, and written informed consent was waived off by means of posters at the implementing institutions. Additionally, this study was in accordance with the STROBE cohort reporting guidelines^9^.

### Procedure

Saline and/or 10% glycerin solution (Glycerol; Chugai Pharmaceutical Co, Tokyo, Japan) with indigo carmine was administered for submucosal injection. For snaring, an electrocautery snare was resected until no remnant existed. For snaring, a 10–33 mm electrocautery snare (SD-210L-10, SD-210U-15, SD-230U-20: SnareMaster and SD-400U-10: SnareMaster Plus; Olympus Medical Systems, Tokyo, Japan, Captivator; Boston Scientific, Marlborough, MA, USA, or 223ADBZX00031000: Dualoop; MEDICO’S HIRATA INC., Osaka, Japan) was used. Any additional treatment (argon plasma coagulation, etc.) was not performed at the time of uniEPMR.

**Figure 1:**
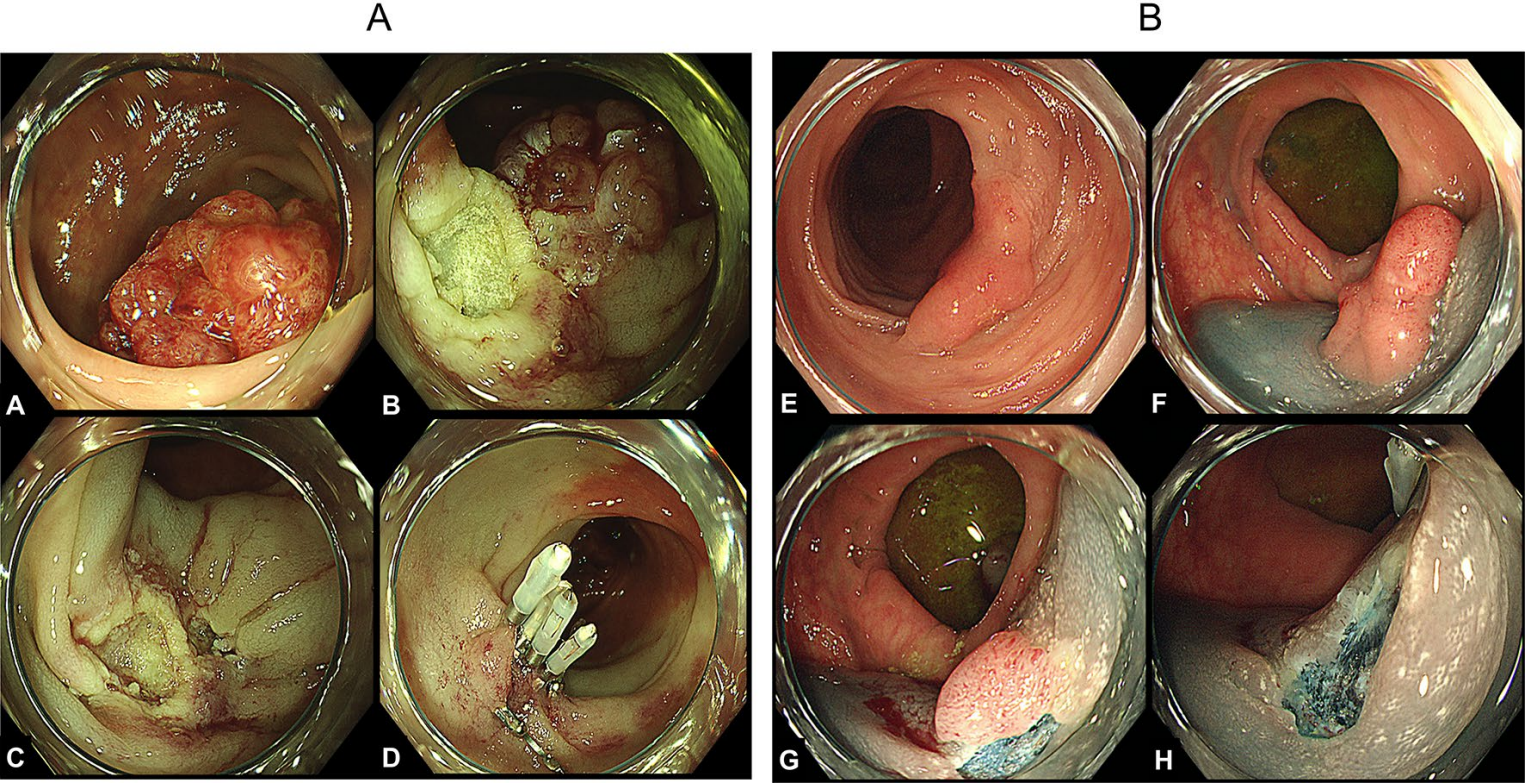
(**A**) A 40-mm lesion located in the sigmoid colon (removed in two pieces). The histopathological diagnosis of this lesion was adenoma. A, Distant image of the lesion. B, First resection. C, Complete resection in the second division. D, Suturing the wound with clips. (**B**) A 15-mm lesion on the folds located in the sigmoid colon (removed in two pieces). The histopathological type was adenoma. E, Distant image of the lesion. F, Local injection just below the lesion. G, First resection. H, Ulcer after the second resection.

Finally, the ulcer was closed with an endoscopic clip at the decision of the endoscopist (Fig. 1A, B). We generally followed the guidelines of the JGES^10,11^ for the management of anticoagulants and antiplatelet agents before and after the procedure.

### Pathological evaluation of the resected specimen

All tissue samples were sent for histological analysis with standard hematoxylin and eosin stain and were evaluated by pathologists at respective institutions separately. Histopathological diagnosis was made by each pathologist based on the general criteria in Japan^12–16^.

### Long-term outcomes

In general, the details on follow-up, such as intervals and methods of assessing for recurrence are as follows. Cases that did not achieve R0 resection (horizontal and vertical margin negative) or did not have surgical indications underwent colonoscopy 3–6 months after endoscopic resection to confirm the presence of residual lesions. Subsequently, annual endoscopic follow-ups were conducted until a clean colon was achieved. All scars were carefully observed using white light, indigo carmine dye combined with white light, or magnification such as narrow-band imaging (NBI). If morphological and/or pathological recurrence was detected, the case was defined as a recurrent case. If the lesion recurred, the recurrence date was collected. In cases without recurrence, the date of the last colonoscopy was collected. Univariate logistic regression models were also established to identify the risk factors of recurrence.

### Evaluation for the experience of endoscopists

The number of experienced cases of EMR and HSP for colorectal lesions in relation to each endoscopist was investigated. The number of experienced cases included all cases performed by each endoscopist. Briefly, lesions < 10 mm and non-neoplastic lesions that were not included in the analysis of lesion features were also calculated as experienced cases. We investigated the number of experienced cases for each endoscopist for the target lesion. The number of cases experienced at the eight facilities other than the three mentioned above was ascertained from the facilities’ databases. Data on the number of other experienced cases were obtained from a questionnaire survey of individual endoscopists to ascertain the number of consecutive cases. The questionnaire survey was also performed to obtain the data at the time of application for the JGES specialty. Additionally, it was determined whether each lesion was resected before or after the qualification of the JGES specialist. In the tabulation of background factors for endoscopists, those who became qualified as JGES specialists during the period covered by this survey and those who were already qualified as specialists at the start of the period covered were counted as the qualified group. The learning curve (LC) of the ratio of uniEPMR and the number of experienced cases for every 10 cases were analyzed.

### Risk factors related to uniEPMR

Univariate and multivariate logistic regression models were established to identify the uniEMR-related risk factors. This analysis was performed from the viewpoint of not only lesions but also endoscopist factors. Lesions on the folds were defined as lesions present, even partially, on any folds of the colon.

### Endpoints

The primary endpoint of this study was the identification of the risk factors of uniEPMR. The secondary endpoints were the identification of risk factors for recurrence after EMR and experienced cases needed to be proficient in en bloc EMR.

### Statistical analysis

Summary statistics [mean ± standard deviation (SD)] were calculated for continuous variables, and the number and proportion of cases were calculated for categorical variables. The clinical outcomes for en bloc resection and uniEPMR were compared using the equal variance two sample t-test or chi-square test. Mixed-effects multivariate logistic regression analysis was performed using lesion size, JGES specialist qualifications, experienced cases, lesions on the folds, non-polypoid lesion, histopathological diagnosis, and lesion localization as explanatory variables; uniEPMR as a response variable; and patients as random effects. For recurrence, the univariate logistic regression and Kaplan–Meier analyses were performed because of the small number of events. The log-rank test was used to compare uniEPMR and en bloc resection in terms of recurrence time. The relationship between the rate of uniEPMR and number of experienced cases was evaluated using the local scatterplot smoother plot (LOESS) with 95% confidence interval (CI). A two-sided significance level of 5% was used in all statistical analyses. All calculations were performed in SAS ver. 9.4 (SAS Institute, Cary, NC, USA).

### Ethics approval statement

This study was approved by the Medical Ethics Board of Chiba University Graduate School of Medicine (protocol no.: 3593), the Medical Ethics Board of Seikei-kai Chiba Medical Center ((protocol no.: CMC2022-5), and the Medical Ethics Board of Chiba Municipal Aoba Hospital (protocol no.:20200901) and was conducted in accordance with the Declaration of Helsinki.

### Patient consent statement

For this was a retrospective cohort study, written informed consent was not obtained. The need for informed consent was waived by the Medical Ethics Board of Chiba University Graduate School of Medicine. Instead, for participation in this study, informed consent was given by means of posters at the implementing institutions. Additionally, this study was in accordance with the STROBE cohort reporting guidelines.

## Results

### Patients and lesions

**Figure 2:**
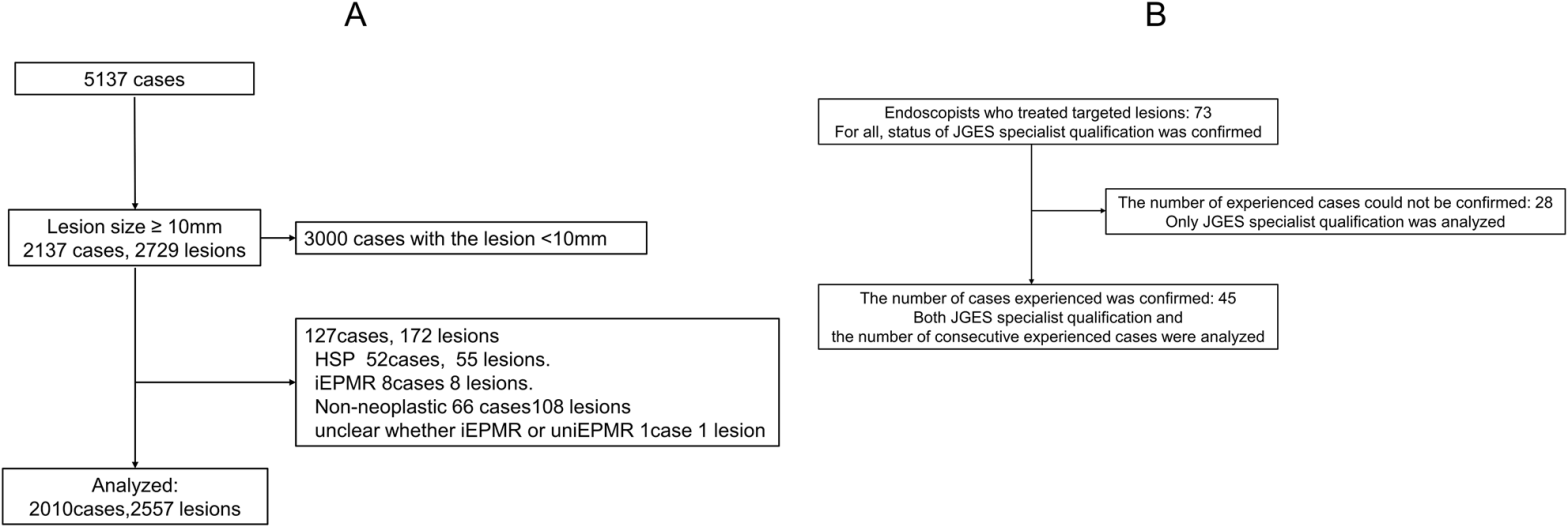
(**A**) Data collection for lesions in three hospitals. HSP, hot snare polypectomy; EPMR, endoscopic piecemeal mucosal resection; iEPMR, intentional EPMR. EMR and HSP cases performed at Chiba University Hospital, Chiba Medical Center, and Chiba Municipal Aoba Hospital from January 2007 to May 2018 were obtained. Overall, 5137 cases were treated. Detailed data were collected for 2137 cases and 2729 lesions ≥ 10 mm. Of these, a total of 127 cases and 172 lesions were excluded. As a result, a total of 2010 cases and 2557 lesions were included in the analysis. (**B**) Data collection on endoscopists. There were 73 endoscopists to be analyzed who were in charge of treating the targeted 2557 lesions. The JGES specialist qualification status of all 73 was confirmed. Of these, 45 could confirm the number of consecutive cases.

Figure 2A shows the study flow of the targeted lesions. A total of 2010 cases and 2557 lesions (adenoma or carcinoma) were analyzed. Table 1 shows the background of 2557 lesions included in the analysis. The overall en bloc resection rate of 2557 lesions was 87.2%. The lesion size (mean ± SD) was 14.3 ± 5.4 mm.

**Table 1:** Characteristics of the resected lesions.

	Patients with lesions for analysis
	(n = 2557)
Lesion size (mm) mean ± SD	14.3 ± 5.4
Location of lesions, n (%)	
Cecum	176 (6.9)
Ascending colon	476 (18.6)
Transverse colon	402 (15.7)
Descending colon	172 (6.7)
Sigmoid colon	1020 (39.9)
Rectum	309 (12.1)
Uncertain	2 (0.1)
Morphology of lesions, n (%)	
Polypoid (Is, Ip)	1914 (74.9)
Non-polypoid (others)	643 (25.1)
Histopathological diagnosis, n (%)	
Adenoma	1955 (76.5)
IMC	457 (17.9)
SM-invasive cancer	145 (5.7)
Pattern of resection, n (%)	
en bloc EMR	2230 (87.2)
uniEPMR	327 (12.8)
Observation period (days), mean ± SD	
No recurrence	621.9 ± 880.5
Recurrence	669.8 ± 464.3

SD, standard deviation; EMR, endoscopic mucosal resection; IMC, intramucosal carcinoma; SM, submucosal layer; uniEPMR, unintentional EPMR.

### Data on endoscopists

Figure 2B shows the data collection flow for endoscopists. There were 73 endoscopists to be analyzed who were in charge of treating the targeted 2557 lesions. The JGES specialist qualification status of all 73 endoscopists was confirmed. Of these, 45 could confirm the number of consecutive cases. For the number of experienced cases, endoscopist data were obtained from serial case data for all treated lesions at the three facilities where targeted lesions were analyzed. For other 8 centers where 45 endoscopists had belonged, a questionnaire survey was performed to obtain consecutive experienced cases.

### Clinical outcomes of EMR

The en bloc resection rates by lesion size were as follows: 90.7% for 10–19 mm, 77.1% for 20–29 mm, 43.6% for ≥ 30 mm, and 87.2% overall. Table 2 shows the results of endoscopic treatment of the 2557 lesions included in the analysis. The recurrence rates of uniEPMR and en bloc resection were significantly different (2.8% vs. 0.3%, p < 0.0001).

**Table 2:** Comparison of clinical outcomes between en bloc EMR and unintentional EPMR.

	uniEPMR	en bloc EMR	p-value†
	(n = 327)	(n = 2230)	
Non-R0 resection, n (%)	**–**	259 (11.6)	N/A
Recurrence, n (%)	9 (2.8)	6 (0.3)	< 0.0001^†^
Adverse event, n (%)			
Postoperative bleeding	12 (3.7)	48 (2.2)	0.0933^†^
Intraoperative perforation	2 (0.6)	3 (0.1)	0.0691^†^
Delayed perforation	1 (0.3)	1 (0.04)	0.116^†^
Lesion size (mm) mean ± SD	18.0 ± 8.0	13.7 ± 4.7	< 0.0001^††^
Location of lesions, n (%)			
Cecum	45 (13.8)	131 (5.9)	< 0.0001^†^
Ascending colon	75 (23.0)	401 (18.0)	
Transverse colon	65 (19.9)	337 (15.1)	
Descending colon	21 (6.4)	151 (6.8)	
Sigmoid colon	76 (23.3)	944 (42.4)	
Rectum	44 (13.5)	265 (11.9)	
Uncertain	1	1	
Morphology of lesions, n (%)			
Polypoid (Is, Ip)	162 (49.5)	1752 (78.6)	< 0.0001^†^
Non-polypoid (others)	165 (50.5)	478 (21.4)	
Number of lesions on the folds, n (%)	134 (41.0)	312 (14.0)	< 0.0001^†^
Number of non-polypoid lesions, n (%)	165 (50.5)	478 (21.4)	< 0.0001^†^

SD, standard deviation; EMR, endoscopic mucosal resection; EPMR, endoscopic piecemeal mucosal resection; uniEPMR, unintentional EPMR; N/A, not applicable.

IMC, intramucosal carcinoma; SM, submucosal layer.

^†^Chi-square test.

^††^Equal variance two sample t-test.

### LC of endoscopists

**Figure 3:**
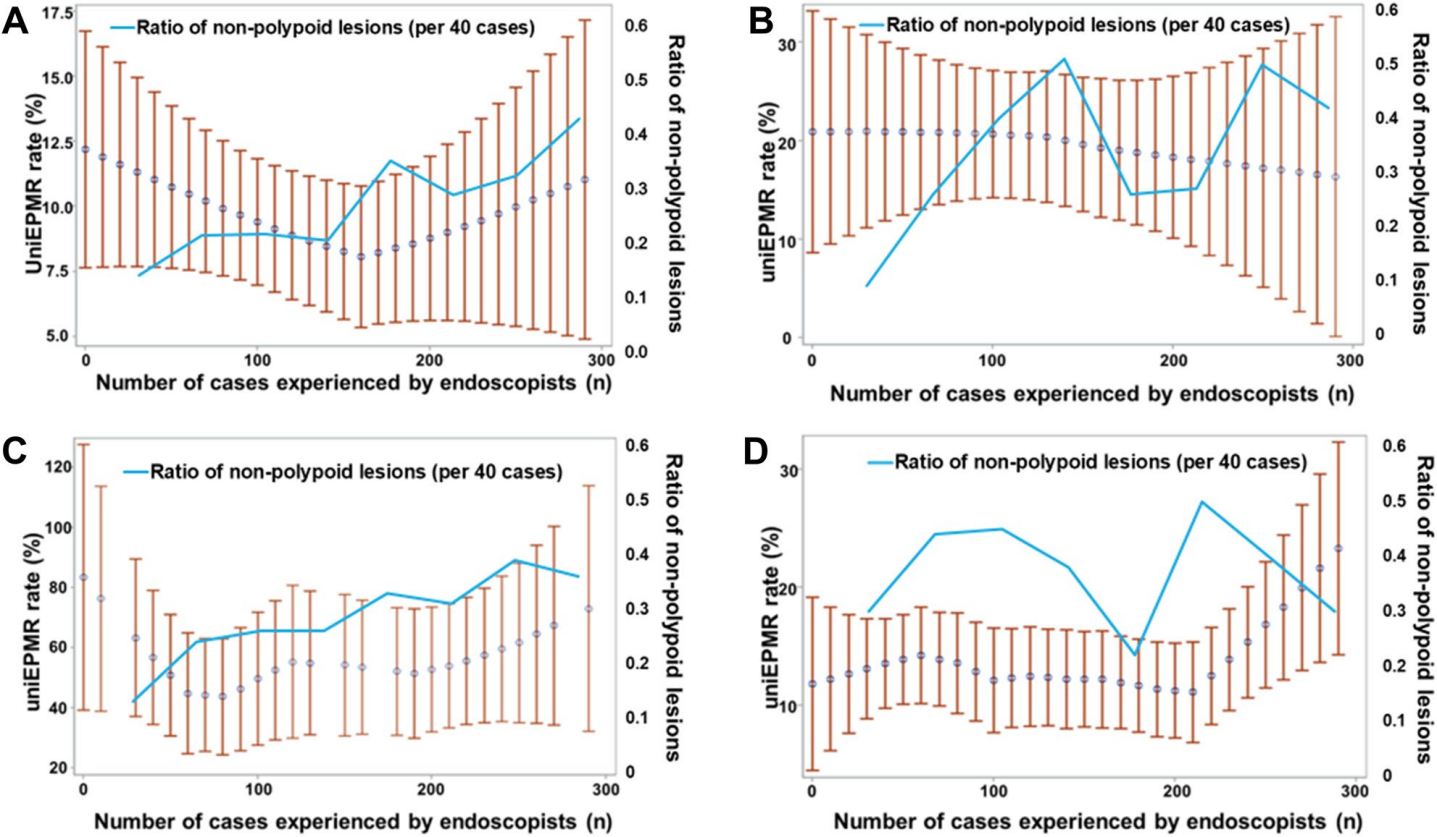
Relationship between the uniEPMR rate and the number of cases experienced (by lesion size). The mean of each endoscopist’s uniEPMR rate is plotted by lesion size (LOESS regression). The vertical axis shows the uniEPMR rate, and the horizontal axis shows the number of experienced cases. The blue circle represents the fitted LOESS curve, and the error bar represents 95% confidence intervals (generally per 10 cases). The same plot was also combined with the percentage of non-polypoid lesions treated according to the number of experienced cases (every 40 cases). This percentage was indicated by a light blue broken line, with the numbers displayed on the vertical axis on the right. (**A**) For lesion sizes 10–19 mm, the uniEPMR rate decreases until about 160 cases, but then increases. (**B**) For lesion sizes 20–29 mm, the uniEPMR rate generally decreased with increasing case experience. (**C**) The uniEPMR rate for lesion sizes ≥ 30 mm was originally high, but the uniEPMR rate did not generally decrease with increasing experience. (**D**) The relationship between the uniEPMR rate and the number of experienced cases for all sizes.

Figure 3 shows the LC of endoscopists. For lesion sizes of 10–19 mm, a decreasing trend was found in the percentage of uniEPMR until about 160 cases, after which there was an increasing trend. Among the range of 10–19 mm, the proportion of non-polypoid lesions was significantly higher in the lesions treated by endoscopists with > 160 than ≤ 160 (27.8 vs 20.2%. p = 0.0002, chi-square test). For lesion sizes of 20–29 mm, the uniEPMR rate generally decreased with increasing case experience. The uniEPMR rate for lesion sizes ≥ 30 mm was originally high, but the uniEPMR rate did not generally decrease with increasing experience.

### Risk factors for uniEPMR

Table 3 shows the risk factors for uniEPMR. In univariate analysis, lesion size ≥ 30 mm, lesion on the folds, cecal lesion, non-polypoidal lesion, lesion size of 20–29 mm, transverse colon lesion, ascending colon lesion, SM-invasive cancer, IMC, descending colon lesion, and rectal lesions were significant variables. In the multivariate analysis, lesion size ≥ 30 mm (odds ratio [OR] 11.83, 95% CI 6.80–20.60, p < 0.0001), lesions on the folds (OR 3.39, 95% CI 2.52–4.55, p < 0.0001), non-polypoid lesions (OR 3.06, 95% CI 2.25–4.14, p < 0.0001) were the three risk factors for uniEPMR with the highest OR.

**Table 3:** Univariate and multivariate analyses for uniEPMR.

	Univariate analysis			Multivariate analysis		
	Crude OR	95% CI	p-value†	Adjusted OR	95% CI	p-value†
Lesion size						
20–29 mm vs. 10–19 mm	3.05	2.21–4.21	< 0.0001	2.52	1.82–3.49	< 0.0001
30 mm- vs. 10–19 mm	14.71	7.65–28.29	< 0.0001	11.83	6.80–20.60	< 0.0001
Specialist	0.99	0.77–1.28	0.9503	1.31	0.98–1.76	0.0713
Experienced cases						
60–150 cases vs. 0–50 cases	1.02	0.64–1.64	0.9229	0.68	0.41–1.14	0.1462
160–250 cases vs. 0–50 cases	0.71	0.43–1.16	0.1727	0.43	0.25–0.74	0.0025
260 cases vs. 0–50 cases	1	0.65–1.54	0.9825	0.75	0.47–1.22	0.2535
Lesions on the folds	4.79	3.38–6.79	< 0.0001	3.39	2.52–4.55	< 0.0001
Non-polypoid lesion	4.37	3.08–6.20	< 0.0001	3.06	2.25–4.14	< 0.0001
Histological diagnosis						
IMC vs. adenoma	1.97	1.45–2.67	< 0.0001	1.62	1.15–2.29	0.0058
SM-invasive cancer vs. adenoma	2.05	1.27–3.31	0.0034	1.63	0.95–2.81	0.0765
Localization of the lesions						
C vs. S	4.63	2.88–7.45	< 0.0001	2.72	1.64–4.51	0.0001
A vs. S	2.43	1.68–3.54	< 0.0001	1.49	0.98–2.27	0.0592
T vs. S	2.54	1.71–3.77	< 0.0001	1.77	1.16–2.71	0.008
D vs. S	1.79	1.04–3.10	0.0363	1.5	0.81–2.78	0.1977
R vs. S	2.1	1.38–3.20	0.0005	2.05	1.31–3.20	0.0017

OR, odds ratio; CI, confidence interval; uniEPMR, unintentional EPMR; IMC, intramucosal carcinoma; SM, submucosal layer; C, cecum; A, ascending colon; T, transverse colon; D, descending colon; S, sigmoid colon; R, rectum.

^†^Logistic regression analysis.

Among lesions ≥ 30 mm, the proportion of Ip morphology lesions was 32.1% of the total. For lesions ≥ 30 mm, the uniEPMR rate was 15.4% for Ip lesions, whereas the uniEPMR rate was 72.7% for non-Ip lesions, and the uniEPMR rate was significantly lower for Ip lesions (p < 0.001, chi-square test).

## Discussions

To the best of our knowledge, this study was the first that focused on uniEPMR and considered not only lesion factors but also endoscopist factors. Regarding lesion factor, ≥ 30-mm lesions were the most associated risk factor. According to previous reports, the EPMR rates were 22% for 20–29-mm lesions, 70.6% for 30–39-mm lesions, and 82.6% for ≥ 40-mm lesions^17,18^. In this study, the uniEPMR rates by lesion size were comparable to previous data. On the contrary, for lesions ≥ 30 mm, the uniEPMR rate of Ip lesions was significantly lower than that of non-Ip lesions. This suggests that en bloc EMR may be indicated for Ip lesions, even if the lesion is ≥ 30 mm.

For large lesions, it may be difficult to confirm that the lesion is firmly contained in the snare or the lesion may be dislodged from the snare by re-snaring, resulting in piecemeal resection. On the contrary, a high en bloc resection rate of 83.5–91.5% was reported for endoscopic submucosal dissection (ESD) in a cohort of patients with large lesions ≥ 20 mm, indicating its superiority to EMR^6,19,20^. For large lesions (≥ 30 mm), ESD was considered a better modality than EMR.

Lesions on the folds have not been well pointed out for risk factors of EPMR so far. The lesion on the fold is originally convex toward the lumen, and the demarcation of the far end is difficult to see even after submucosal injection. When non-polypoid lesions are injected, the lesions become even more flat or depressed than their original form, making it difficult to snare. The cecum often has a large lumen, and the actual lesion size is prone to be larger than the size based on endoscopic observation. Another characteristic of cecal lesions is that they tend to have large amounts of fat and fiber in the SM, making it difficult to achieve distension by local injection. These factors appeared to be hazards to achieving en bloc resection. Recently, the superiority of the en bloc resection rate of underwater EMR (UEMR) for colorectal lesions ranging from 10 to 20 mm over conventional EMR has been reported (89% vs 75%)^21^. Lesions with these risk factors should be considered for other modalities such as UEMR or ESD.

Regarding LC, after the uniEPMR rate decreased to 160 experienced cases, the rate increased in 170 cases and later in lesions of 10–19 mm. The reasons for this were considered as follows. The ratio of non-polypoid lesions in ≥ 170 experienced cases among lesions of 10–19 mm is significantly higher than that in ≤ 160. In other words, endoscopists tended to perform EMR or HSP for difficult lesions, as they experienced more cases. Additionally, lesions 10–19 mm were the majority, accounting for over 80% of all lesions. The uniEPMR rate after 170 cases for 10–19-mm lesions appeared to have affected the increase in the uniEPMR rate in the LC for all sizes of lesions. On the contrary, for 20–29-mm lesions, the uniEPMR rate tended to decrease, as the number of cases experienced by endoscopists increased. No association between the number of experienced cases and the rate of uniEPMR was found for ≥ 30-mm lesions, and it was considered that piecemeal resection could occur even if experienced endoscopists performed the procedure. Considering the lesion factors together, EMR is not indicated for ≥ 30-mm lesions from the viewpoint of en bloc resection. Bhurwal et al. reported that 100 EMR procedures for large non-polypoid colorectal neoplasia are required to achieve a plateau phase for the overall rates of residual neoplasia and incomplete EMR for those lesions^19^. Considering the results of this study, including polypoid and non-polypoid lesions, approximately 160 cases (where the unintentional rate among lesions of 10–19 mm was the lowest on the LC) were considered a guideline for achieving en bloc resection. In fact, endoscopists’ experience of 160–250 cases (vs. 0–50 cases) was a significantly low-risk factor for uniEPMR on multivariate analysis. On the other hand, the same factor did not show the same result on univariate analysis. To determine whether 160 cases are true guideline for achieving en bloc resection, the further investigation would be required. In this study, we also examined the presence or absence of JGES qualifications but found no clear relationship between specialist qualifications and uniEPMR rate. This may be because the timing of specialty qualifications was left to the discretion of each endoscopist and therefore did not necessarily correlate positively with the number of cases experienced.

The recurrence rate of uniEPMR in this study was 2.8%, which was lower than that in previous reports (7.1–63.6%)^20,22–25^. The recurrence rate could be low to some extent if endoscopist resects until no morphological remnant is found. In this study, the recurrence rate of en bloc EMR was 0.3%, which was significantly lower than that of uniEPMR. Previous reports have shown that the recurrence rate of uniEPMR is higher than that of en bloc EMR (0.0–9.1%)^24–26^. This indicates that endoscopic en bloc resection is crucial for achieving lower recurrence.

This study had several limitations. First, this was a retrospective study. To obtain an accurate LC, a prospective study based on the fixed protocol is needed. Second, the number of cases experienced by each endoscopist other than three major institutes was based on a questionnaire survey, including HSP and EMR. Third, this study was a multi-center retrospective study, the exact number of divisions for uniEPMR was unknown. Fourth, there is no uniform criterion for the indication of EMR and ESD for large-sized lesions or non-polypoid lesions.

In conclusion, 160 experienced cases seemed to be the minimum case number needed to be proficient in en bloc EMR. Additionally, while lesion sizes of 10–29 mm are considered suitable for EMR, ≥ 30-mm lesions are not suitable for en bloc EMR from the perspective of both lesion and endoscopist factors. To demonstrate the validity of these conclusions, a prospective study with consecutive cases would be necessary.

## Data Availability

The datasets used and/or analyzed during the current study available from the corresponding author on reasonable request.

## Abbreviations

CRC: Colorectal cancer
EMR: Endoscopic mucosal resection
HSP: Hot snare polypectomy
CP: Cold polypectomy
HP: Hyperplastic polyp
SSL: Sessile serrated lesion
TSA: Traditional serrated adenoma
IMC: Intramucosal carcinoma
SM: Submucosal layer
EPMR: Endoscopic piecemeal mucosal resection
iEPMR: Intentional EPMR
uniEPMR: Unintentional EPMR
OR: Odds ratio
CI: Confidence interval
N/A: Not applicable
LC: Learning curve
JGES: Japan Gastroenterological Endoscopy Society
